# Bufalin targeting CAMKK2 inhibits the occurrence and development of intrahepatic cholangiocarcinoma through Wnt/β-catenin signal pathway

**DOI:** 10.1186/s12967-023-04613-6

**Published:** 2023-12-11

**Authors:** Huhu Zhang, Xiaolei Dong, Xiaoyan Ding, Guoxiang Liu, Fanghao Yang, Qinghang Song, Hongxiao Sun, Guang Chen, Shuang Li, Ya Li, Mengjun Wang, Tingting Guo, Zhe Zhang, Bing Li, Lina Yang

**Affiliations:** 1 Department of Genetics and Cell Biology, Basic Medical College, Qingdao University, Qingdao, 266071 China; 2 Department of Clinical Laboratory, Weifang People’s Hospital, 151, Guangwen Street, Weifang, 261041 China; 3 Health Science Center, Qingdao University, Qingdao, 266071 China; 4 Heart Center, Women and Children’s Hospital, Qingdao University, 6, Tongfu Road, Qingdao, 266034 China; 5 Department of Hematology, The Affiliated Hospital of Qingdao University, Qingdao, 266003 China

**Keywords:** Intrahepatic cholangiocarcinoma, Bufalin, CAMKK2, Ca^2+^, ANXA2, Mitochondrial dysfunction, Proliferation, Migration

## Abstract

**Background:**

Intrahepatic cholangiocarcinoma (ICC) accounts for about 15% of primary liver cancer, and the incidence rate has been rising in recent years. Surgical resection is the best treatment for ICC, but the 5-year survival rate is less than 30%. ICC signature genes are crucial for the early diagnosis of ICC, so it is especially important to find its signature genes and therapeutic drug. Here, we studied that bufalin targeting CAMKK2 promotes mitochondrial dysfunction and inhibits the occurrence and metastasis of intrahepatic cholangiocarcinoma through Wnt/β-catenin signal pathway.

**Methods:**

IC50 of bufalin in ICC cells was determined by CCK8 and invasive and migratory abilities were verified by wound healing, cell cloning, transwell and Western blot. IF and IHC verified the expression of CAMKK2 between ICC patients and normal subjects. BLI and pull-down demonstrated the binding ability of bufalin and CAMKK2. Bioinformatics predicted whether CAMKK2 was related to the Wnt/β-catenin pathway. SKL2001, an activator of β-catenin, verified whether bufalin acted through this pathway. In vitro and in vivo experiments verified whether overexpression of CAMKK2 affects the proliferative and migratory effects of ICC. Transmission electron microscopy verified mitochondrial integrity. Associated Ca^2+^ levels verified the biological effects of ANXA2 on ICC.

**Results:**

It was found that bufalin inhibited the proliferation and migration of ICC, and CAMKK2 was highly expressed in ICC, and its high expression was positively correlated with poor prognosis.CAMKK2 is a direct target of bufalin, and is associated with the Wnt/β-catenin signaling pathway, which was dose-dependently decreased after bufalin treatment. In vitro and in vivo experiments verified that CAMKK2 overexpression promoted ICC proliferation and migration, and bufalin reversed this effect. CAMKK2 was associated with Ca^2+^, and changes in Ca^2+^ content induced changes in the protein content of ANXA2, which was dose-dependently decreasing in cytoplasmic ANXA2 and dose-dependently increasing in mitochondrial ANXA2 after bufalin treatment. In CAMKK2 overexpressing cells, ANXA2 was knocked down, and we found that reversal of CAMKK2 overexpression-induced enhancement of ICC proliferation and migration after siANXA2.

**Conclusions:**

Our results suggest that bufalin targeting CAMKK2 promotes mitochondrial dysfunction and inhibits the proliferation and migration of intrahepatic cholangiocarcinoma through Wnt/β-catenin signal pathway. Thus, bufalin, as a drug, may also be used for cancer therapy in ICC in the future.

**Supplementary Information:**

The online version contains supplementary material available at 10.1186/s12967-023-04613-6.

## Introduction

Cancer is an overarching term for more than 100 unique types of malignant tumors in different organs [1, 2]. Hepatocellular carcinoma is divided into primary and secondary hepatocellular carcinoma. Primary liver malignancies originate in the epithelial or mesenchymal tissues of the liver [3, 4]. Intrahepatic cholangiocarcinoma (ICC) is a type of primary liver tumor, and surgical resection is still considered the only treatment for ICC [5]. ICC has a high incidence, high malignancy, and poor prognosis in China and Southeast Asia [6]. Due to its high mortality rate, ICC remains a fatal malignancy for most patients [7] with a five-year survival rate of less than 30%. There are no obvious symptoms in the early stage of ICC, and liver masses are often found incidentally when imaging tests are performed in the presence of abnormal liver function [8]. Risk factors for the development of ICC include chronic cholangitis, chronic inflammatory bowel disease, parasitic infections, chemical carcinogens, genetic factors, biliary cirrhosis, alcoholic liver disease, and non-specific cirrhosis [9]. In addition, hepatitis viruses have been found to be strongly associated with ICC [10]. Mechanisms by which key gene mutations and aberrant signalling pathways such as TP53, KRAS, ARID1A, IDH1/2 mutations and FGFR gene fusions drive the development of ICC [11]. There is a correlation between ICC development and aberrantly expressed non-coding RNAs in tumor cells, and miR-370 in ICC inhibits the proto-oncogene MAP3K8 [12]. For the past decade, the treatment of ICC has typically used gemcitabine and cisplatin [13]. However, drug insensitivity or rapidly developing chemoresistance resistance may lead to poor outcomes. Therefore, it is particularly important to identify new therapeutic agents and relevant targets in ICC.

Intracellular calcium homeostasis is frequently disturbed during tumorigenesis and progression. Intracellular calcium ion (Ca^2+^) is a common second messenger that regulates a variety of cellular pathophysiological processes such as cell proliferation and migration [14]. Recent studies have shown that the remodeling of Ca^2+^ signaling is associated with cancer development, progression, and metastasis [15]. Ca^2+^ channels, transporters and pumps regulate calcium movement, and alterations expression/activity of Ca^2+^ signaling components are associated with many cancer cell activities [15]. Calcium/calmodulin-dependent protein kinase kinase 2 (CAMKK2) is a serine/threonine protein kinase that belongs to the Ca^2+^/calmodulin-dependent protein kinase subfamily. CAMKK2 has been reported to be highly expressed in prostate cancer [16], hepatocellular carcinoma [17], ovarian cancer [18] and gastric cancer [19]. Furthermore, CAMKK2 has not been reported in ICC.

Annexin A2 (ANXA2), a member of the ANXA protein family, is a 36 kDa calcium-dependent phospholipid-binding protein that is commonly expressed in a variety of eukaryotic cells [20]. As a multifunctional protein, ANXA2 can interact with a variety of ligands and affect a variety of cellular processes, such as Ca^2+^ transport, endocytosis, extracellular processes, tissue remodeling, angiogenesis, and immune regulation [21, 22]. Aberrant expression of ANXA2 is observed in a variety of malignancies, including hepatocellular carcinoma, and plays a key role in tumor formation and progression by regulating cell proliferation, apoptosis, adhesion, invasion, metastasis, and tumor neovascularization [23, 24]. However, the role of ANXA2 in ICC has not been reported in studies.

Bufalin, an active monomer extracted from toadstool, is a potent anticancer drug and has a variety of biological activities [25]. Previous studies have shown that bufalin inhibits cancer proliferation, invasion, and migration by inducing different cell-death mechanisms, such as apoptosis, necrosis, and autophagy [26]. Bufalin regulates metastasis formation by blocking angiogenesis and reducing cancer cell stemness [27]. Bufalin also affects the immune microenvironment by reversing various drug resistance mechanisms [28]. These studies support bufalin as a very promising new anti-cancer drug candidate that has not previously been studied for the treatment of ICC.

So far, the studies on disease prevention and potential drug-targeted therapy for ICC is unclear and deserves further study. This study investigated the effect and mechanism of CAMKK2 overexpression on the malignant phenotype of ICC cells as well as the effect of bufalin on ICC in vitro and in vivo.

## Materials and methods

### Reagents

Bufalin were provided from Selleck (Houston, Texas, USA). Phosphate buffer (PBS), trypsin, penicillin, streptomycin, 1640 and DMEM medium were obtained from BI (Xuzhou, China). Protease inhibitors and the Cell Counting Kit-8 assay (CCK-8) were purchased from Thermo Fisher (Waltham, Massachusetts, USA). N-cadherin and Vimentin antibodies for western blotting analysis were provided by yazyme biology (Shanghai, China). Anti-CaMKK2 antibodies were provided by Proteintech (Chicago, USA). Anti-β-catenin, anti-^Ser33^P-β-catenin, anti- ANXA2 antibodies and anti-cyclin D1 antibodies were purchased from AB clonal (Shanghai, China).

### Cell lines

Human intrahepatic cholangiocarcinoma cell lines (HCCC-9810, QBC-939 and RBE) and Normal liver cell line (L02) were purchased from Yubo Biology (Shanghai, China). All cells were cultured in 1640 medium containing 10% fetal bovine serum, 100 μg/mL streptomycin and 100 μg/ mL penicillin in a humidified incubator with 5% CO_2_ at 37 °C. The expression level of CAMKK2 in ICC was verified. Three kinds of ICC cells were compared with normal cells L02, and it was found that CAMKK2 was highly expressed in ICC. When CAMKK2 was overexpressed in ICC cells, we selected two types of cells with low ICC expression of CAMKK2 (HCCC-9810 and QBC-939) for verification. In addition, as it has been reported that HCCC-9810 and RBE cells could not have the conditions for tumor formation in nude mice, we select QBC-939 for the verification of the mouse experiment.

### Cell viability assay

L02, HCCC-9810, RBE and QBC-939 Cells were inoculated in 96-well plates and treated with bufalin for 24 h, 48 h and 72 h, and then cell viability was measured according to CCK8. Cell viability was determined by absorbance measurement at 450 nm by spectrophotometer [29].

### Cell plate cloning

HCCC-9810, RBE and QBC-939 Cells were cultured in 80% to 90% of 6-well petri dishes. Replace the medium with fresh medium containing bufalin at a certain concentration. Add 1 ml of 4% paraformaldehyde into each well to fix the cells for 20 min, add appropriate amount of crystal violet cells into the well tostain for 15 min, and count the clone colonies [30].

### Wound healing

Two to three horizontal lines were evenly drawn on the back of the 6-well plate, and 2 mL HCCC-9810, RBE and QBC-939 cells with a cell density of 5.0 × 10^5^/mL were added. The cells were cultured for 24 h. When the cell density reached 95%, the cells were scratched along the underside with the tip of 200μL and cleaned with PBS buffer for 2–3 times. The culture was continued and photographs were taken at 24 h and 48 h respectively [29].

### Transwell

For cell migration assay, transwell inserts with 6.5 mm polycarbonate membrane and 8.0 mm holes were used. Then 1 × 10^4^ HCCC-9810, RBE and QBC-939 cells were placed in the upper chamber, and the number of cells migrating to the lower chamber was counted after a period of incubation. After incubation for a period of time, the cell motility was observed. The cells were placed in basal medium and the primer was serum-containing medium. After incubation at 37 °C for 2 days, wipe the top chamber with cotton wool to remove non-migration cells. The cells under the membrane were fixed with paraformaldehyde for 30 min and then stained with 0.1% crystal violet. The number of cells were observed under the microscope. Each experiment was repeated three times and averaged [29].

### Preparation of the siRNA-mediated knockdown

Silenced RNA (siRNA) vector targeting ANXA2 was constructed by OBiO Technology Company (Shanghai, China). The RNAi target sequence is shown below: ANXA2-RNAi: 5′-TGTGTGGTGGAGATGACTGA-3′. The old medium in the orifice plate was drained and cleaned with pre-warmed PBS to remove the residual serum. The basic medium without serum and double antibody (6-well plate 2 mL/well) was replaced and cultured in a 37 °C incubator. At the same time, the transfer dye solution was prepared with sterilized EP tube. Take the amount of one well in a six-well plate as an example: Liquid A: Dilute 5 μg negative control with 250 μL Opti-MEM; liquid B: dilute 5 μL lipo2000 with 250 μL Opti-MEM. Si UBA3 as above. Gently mix liquid A and liquid B, respectively, and let it stand at room temperature for 5 min. Add liquid B to liquid A, gently mix it, leave at room temperature for 20 min, and then add it to the 6-well plate [31].

### Western blotting

HCCC-9810, RBE and QBC-939 Cells (treated under different conditions for 48 h) were lysed by RIPA containing protease and phosphatase inhibitors, and the proteins were quantitatively analyzed using BCA kit based on SDS-PAGE gel electrophoresis. During western blotting, enhanced fluorescence substrates were used according to the instructions. A chemiluminescence detection system was used for spectral analysis [32]. To observe the ability of cells to proliferate and migrate in ICC, we examined N-Cadherin and Vimentin proteins. To detect the CAMKK2-influenced Wnt/β-catenin signalling pathway, we detected β-catenin, P-β-catenin, and CCND1. In addition, the negative control was the control group, and the groups were positive controls for each other.

### Molecular docking

The 2D structure file of bufalin was downloaded from the Pubchem database (https://pubchem.ncbi.nlm.nih.gov/) and the 3D structure of CAMKK2 was obtained from the Protein Data Bank. For MOL2 fles of TCM, it is recommended to download them using the TCM Systematic Pharmacology Database and Analysis Platform (TCMSP) (https://old.tcmsp-e.com/tcmsp.php). Water molecules were removed, ligands and receptors were separated, polar hydrogens were added, gasteiger charges were calculated, AD4 types were assigned and fusible bonds of small ligands were set to rotatable using Auto Dock Tools 1.5.6 and Py MOL 2.3.4 software. The molecular docking process was performed using Auto Dock Vina 1.1.2. Py MOL 2.3.4 software and the Protein–Ligand Interaction Profler (PLIP) platform (https://projects.biotec.tudresden.de/plip-web/plip/index) were used for visualisation [33]. The PDB number of the 3D structure of CAMKK2 from the PDB database is 6Y3O and the accession number for bufalin from the Pubchem database is 9,547,215.

### Mouse model

Four-week-old female BALB/c nude mice were purchased from Beijing Vital River Laboratory (Beijing, China) and housed in a specific pathogen-free (SPF) environment. All animal experiments were conducted in accordance with the guidelines established by the institutional ethical committee of Qingdao University (Qingdao, China, Code of Ethics: QDU-ACE-2022507). To establish the ICC xenograft model, 5 × 10^6^ QBC-939 cells infected with the lentivirus were subcutaneously injected into the right flank of the mice (n = 4 mice per group). when the average tumor volume reached 100 mm^3^, the mice in the treatment group were intraperitoneally injected with bufalin (1 mg/kg, 100 μl) every two days, while the other groups were injected with the same volume of normal saline (100 μl) for 2–3 weeks. After bufalin treatment, the mice were euthanized, and the tumors and other organs were removed for histological analysis. The tumor volume was calculated using the formula: 1/2 × (length × width^2^), and recorded for analysis [33].

### Immunofluorescence (IF)

Cultured HCCC-9810, RBE and QBC-939 cells (5 × 10^4^) were fixed in 4% paraformaldehyde for 30 min, removed and placed in 0.5% Triton X-100 for 10 min, and then sealed with 100% FBS for 1 h. The fixed cells were mixed with antibodies to CAMKK2, β-catenin, P-β-catenin and cyclin D1 and incubated for 2 h under standard conditions. Subsequently, the DAPI was incubated for 5 min and washed with PBS. Images were captured using an ordinary fluorescence microscope, and ImageJ-win64 software was used to analyze the fluorescence results [29].

### Proteome microarray

The preparation of human protein microarrays and the synthesis of biotin-bufalin are described above. Sealing buffer (1% bovine serum albumin; 0.1% twain 20; TBST) and stirred gently at 25 ℃ for 1 h. Biotin-bufalin was diluted to 10 μM in blocking buffer and incubated at 25 ℃ for 1 h on proteome microarray. The biotin-Bufalin was washed with thiobarbituric acid for 3 times, 5 min each time, and then diluted with Cy3-streptavidin 1:1000 for 1 h at 25 ℃. Then the biotin-bufalin was washed with thiobarbituric acid for 3 times, 5 min each time. The microarrays were dried at 250 ×*g* for 3 min and scanned with a GenePix 4200A microarray scanner (Axon Instruments) and the results were recorded. GenePix Pro-6.0 software was used for data analysis [32] (see Additional file 1).

### Cell calcium content detection

Extract QBC-939 cells and add 150–250 μL of lysate per 1 × 10^6^ cells; grind using a sample grinder until fully lysed. After bulk lysis, centrifuge at 10,000–14,000*g* for 3–5 min at 4 °C, remove the supernatant and place on ice for measurement. Calcium content assay procedures follow the instructions of the Calcium Colourimetric Assay Kit (Beyotime Biotechnology, Cat. # S1063S, China) [34].

### Immunohistochemistry (IHC)

In this study, 150 postoperative specimens of intrahepatic cholangiocarcinoma and 150 tissue specimens of normal liver were collected from the Affiliated Hospital of Qingdao University from 2019 to 2022. Pathological grading was assessed according to Edmondson Steiner grading system. The staining grade of tissue sections and anti CAMKK2 antibody (diluted 1:200) were analyzed in a wet box at 4 ℃ [35].

### Bio-layer interferometry

Superchain Affinity Tips (ForteBio) were pre-wetted with 0.01 M PBS buffer plus 5‰. DMSO (pH 7.4) as a background buffer for fixation. Binding kinetics were monitored using the Octet QKe system from ForteBio. Coupling of 20–50 μg/mL biotinylated proteins established a stable baseline (60 s), and non-specific binding was flushed with PBS buffer (60 s). A new baseline was established by adding 5‰ DMSO (pH 7.4) to 0.01 M PBS buffer. Biotin- as was prepared as serial diluents (2, 10 and 100 μM) and bound to the saturated tips of the target proteins for 60 s, then separated for 300 s in 0.01 M PBS buffer (pH 7.4) with 1 mM BAL and 5‰ DMSO added to the buffer. X Results were recorded and processed with Octet software v7. X From ForteBio [36].

### HE staining

Tissues were fixed in 4% paraformaldehyde at 4 °C for 24 h and then embedded in paraffin. The resulting sections were then cut into 4 µm thick slices using a tissue slicer (RM2235, Leica) and dried for 24 h at room temperature. After dewaxing with xylene and ethanol, paraffin-embedded tumor and normal tissue sections were rehydrated and stained with hematoxylin and eosin. The sections were then viewed and imaged under a microscope (AX8170-02, Olympus) [33].

### Statistical analysis

Data were expressed as mean ± SD of independent experiments repeated three times under the same conditions. Wound Healing and Western blotting data were analyzed by ImageJ-win64 software, and unpaired T test or one-way ANOVA was performed on all data using GraphPad Prism 8.0 and SPSS 17.0 software. P < 0.05 was used to determine that the data was statistically significant.

## Results

### Bufalin inhibited proliferation and migration in ICC cells

**Fig. 1:**
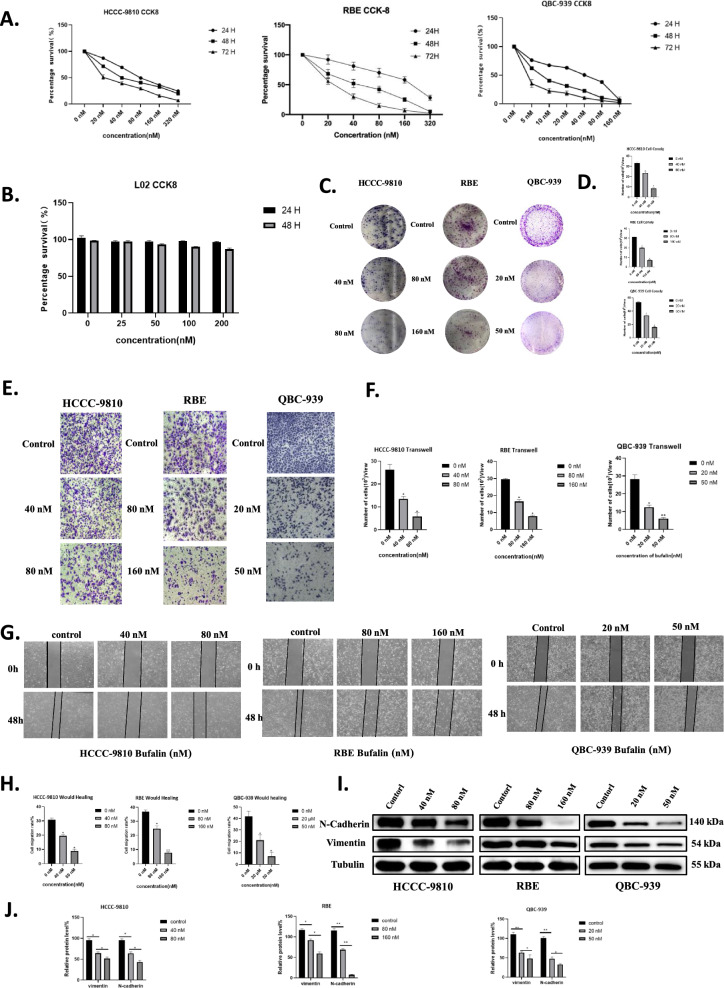
Bufalin inhibited ICC cell viability and metastasis. **A-B** CCK8 assay. In L02, HCCC-9810, RBE, and QBC-939 cells, bufalin resulted in a significant dose-dependent reduction in cell viability compared to controls. Bufalin had no significant effect on the activity of L02 cells. **C** Cell cloning of HCCC-9810, RBE, and QBC-939 cells exposed to bufalin at different concentrations and controls was analyzed. **D** Statistical analysis of cell proliferation capacity in cell cloning experiments. **E** Transwell was analyzed in HCCC-9810, RBE, and QBC-939 cells exposed to bufalin for different time or control. **F** Statistical analysis of the cell migration in the scratch wound healing assays. **G** Wound healing was analyzed in HCCC-9810, RBE, and QBC-939 cells exposed to bufalin for different time or control. **H** Statistical analysis of the cell migration in the scratch wound healing assays. **I** Western blot analysis of expression of metastasis related proteins N-cadherin and Vimentin in HCCC-9810, RBE, and QBC-939 cells exposed to bufalin or control. **J** The quantification of western blot. Protein levels were normalized to tubulin. All results were presented as the mean ± SD (n = 3). *p < 0.05, **p < 0.01

The proliferation of ICC cells was significantly inhibited by different concentrations of bufalin (0, 20, 40, 80, 160, 320 nM), and cell growth was dose- and time-dependent (Fig. 1A). However, there was no significant inhibitory effect on normal hepatocyte L02 cells (Fig. 1B). Cell plate cloning analysis showed that the number of ICC cell colonies decreased as the bufalin concentration increased (Fig. 1C, D). Bufalin hindered the migration and invasion of ICC cells, and the inhibitory effect increased in a dose-dependent manner with the increase of the bufalin concentration (Fig. 1E, F). With the gradual increase of bufalin concentration, the migration rate of ICC cells gradually decreased (Fig. 1G, H). Compared with the NC group, as the bufalin concentration increased, the protein levels of N-cadherin and vimentin in HCCC-9810, RBE, and QBC-939 cells were continuously reduced (Fig. 1I, J). Therefore, bufalin can inhibit the proliferation and migration of ICC cells.

### CAMKK2 was a direct interacting protein of bufalin.

**Fig. 2:**
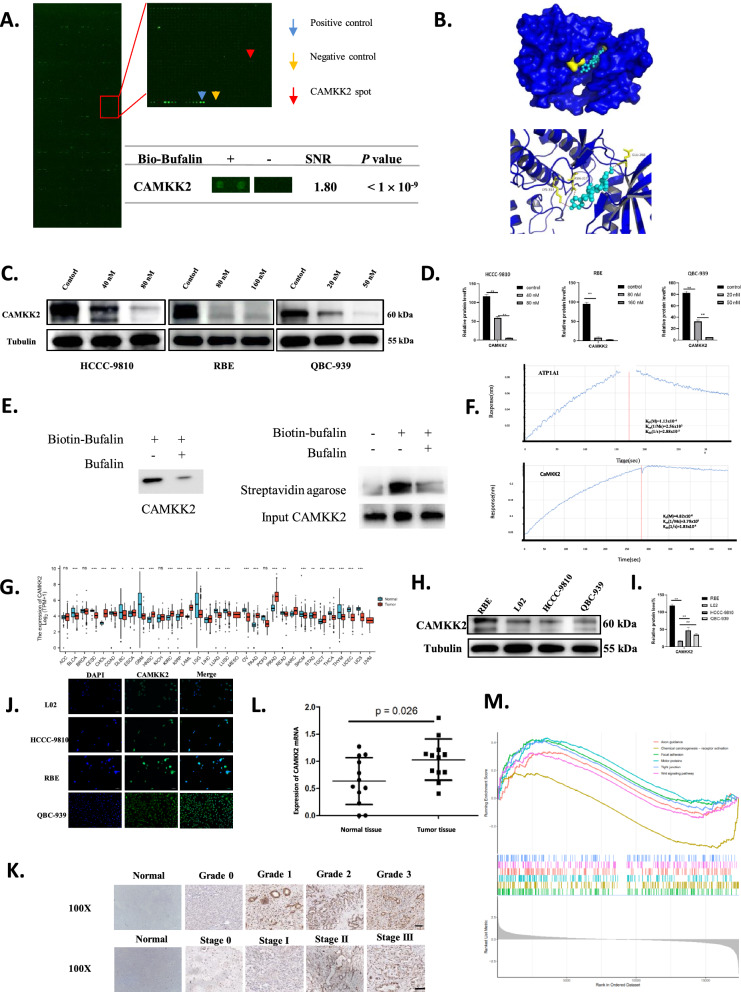
CAMKK2 is a direct interacting protein of bufalin and highly expressed in ICC. A Representative proteome microarrays results. Bufalin–protein interaction was detected (bule boxes) between bufalin and CAMKK2 by red fluorescence. Representative images of protein microarray showed positive control (blue arrow), negative control (yellow arrow) and CAMKK2 spot (red arrow) on the enlarged images. **B** Molecular docking predicted the potential binding sites of bufalin and CAMKK2. **C** Western blot analysis of CAMKK2 expression in HCCC-9810, RBE, and QBC-939 cells treated with different concentrations bufalin. **D** The quantification of western blot. Protein levels were normalized to tubulin. **E** Bufalin is labeled with biotin, pull down and verify that CAMKK2 is the target of bufalin. **F** CAMKK2 has stronger binding force than ATP1A1. **G** Pan-cancer analysis CAMMK2 was highly expressed in ICC, with red representing tumors and blue representing normal tissues.** H** Western blot analysis of CAMKK2 expression in HCCC-9810, RBE, L02 and QBC-939 cells. **I** The quantification of western blot (H). **J** The expression and cell localization of CAMKK2 in HCCC-9810, RBE, L02 and QBC-939 cells were analyzed by IF. **K** The expression level of CAMKK2 in tumor tissues and normal tissues. **L** mRNA expression levels of CAMKK2 in tumor tissue and normal tissue. **M** In the TCGA database, the top 6 signaling pathways related to CAMKK2 were enriched. All results were presented as the mean ± SD (n = 3). *p < 0.05, **p < 0.01

To determine the mechanism of action of bufalin in inhibiting the occurrence and development of ICC, we used human proteomic microarrays to screen for proteins that directly interact with bufalin. We identified 274 candidate proteins with signal-to-noise ratios (SNR) greater than 1.8. Interestingly, CAMKK2 was identified as a promising candidate protein (Fig. 2A). Molecular docking was used to predict the potential binding sites between bufalin and CAMKK2, resulting in binding energies of – 7.9 kcal/mol (Fig. 2B). The expression of CAMKK2 decreased in a dose-dependent manner with increases in the bufalin concentration (Fig. 2C, D). According to the results of the pull-down assay, bufalin could bind to CAMKK2 (Fig. 2E). Interestingly, ATP1A1 is known to be a direct binding site for bufalin [37]. We used bio-layer interferometry (BLI) to determine the binding force between ATP1A1 and bufalin and the binding affinity between CAMKK2 and bufalin. The results showed that the binding force between CAMKK2 and bufalin was much larger than that between ATP1A1 and bufalin (Fig. 2F). Moreover, CAMKK2 was the target of bufalin. These findings suggest that CAMKK2 is a direct target of bufalin.

### CAMKK2 is highly expressed in ICC

Pan-cancer analysis showed that CAMKK2 was significantly overexpressed in ICC (Fig. 2G). The expressions of CAMKK2 in HCCC-9810, RBE, and QBC-939 were higher than L02 (Fig. 2H, I). CAMKK2 was localized in the cytoplasm, and the expression of ICC was significantly higher than L02 (Fig. 2J). Meanwhile, CAMKK2 was mainly localized in the cell membrane and cytoplasm. The expression of CAMKK2 increased with the increased of grade and stage. In addition, the expressions level of CAMKK2 in tumor tissues was significantly higher than in adjacent tumors (Fig. 2K). The mRNA expression of CAMKK2 in ICC patients was significantly higher than in normal patients (Fig. 2L). Gene expression data from the TCGA-CHOL database for ICC and normal tissues, and then performed differential expression analysis using the limma package to screen out differentially expressed genes (DEGs). Functional enrichment analysis of DEGs and found that CAMKK2 was related to the Wnt signaling pathway. Single-gene enrichment analysis of CAMKK2 with GSEA software, which further confirmed its association with the Wnt signaling pathway (Fig. 2M, Table 1). Taken together, these results suggest that CAMKK2 is highly expressed in ICC.

**Table 1:** The result of GSEA

ID	EnrichmentScore	NES	Pvalue	Rank
Focal adhesion	197	0.420873	0.000227	0.003199
Chemical carcinogenesis—receptor activation	194	-0.3572	0.01305	0.051221
Motor proteins	182	0.433368	0.000225	0.003199
Axon guidance	180	0.333757	0.029279	0.092883
Wnt signaling pathway	167	0.326993	0.04387	0.120944
Tight junction	162	0.413593	0.000445	0.003438
Hepatitis C	154	0.363179	0.014078	0.054337
Hippo signaling pathway	150	0.384154	0.004019	0.018993
Phospholipase D signaling pathway	142	0.371791	0.011328	0.047698
Signaling pathways regulating pluripotency of stem cells	140	0.3459	0.037119	0.107987

### Bufalin inhibited Wnt/β-catenin signal pathway in ICC cells

**Fig. 3:**
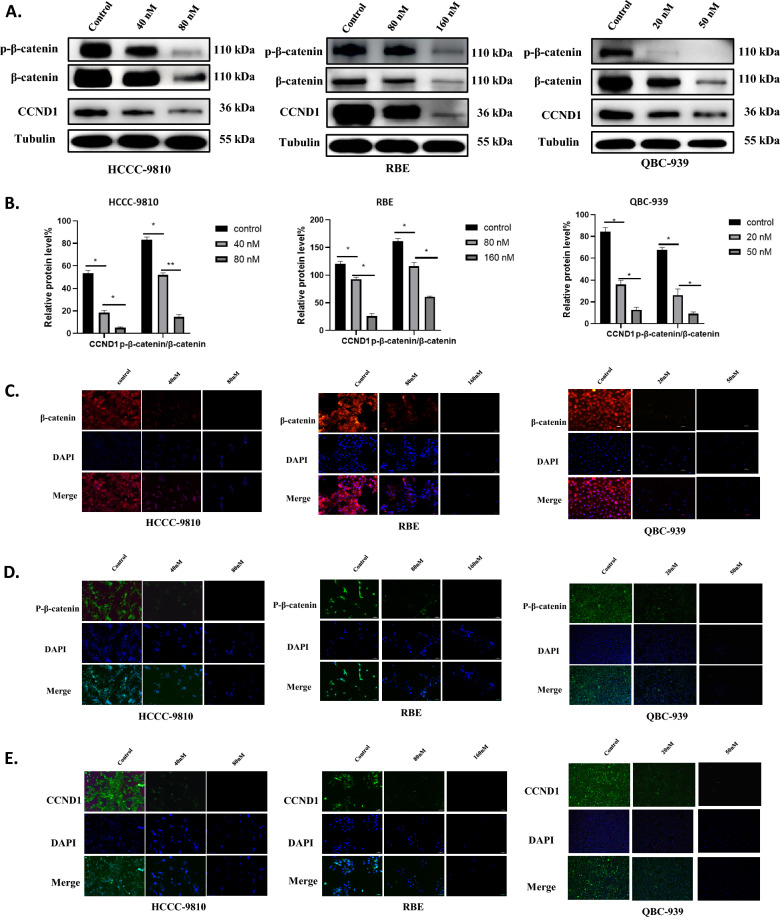
Bufalin inhibited Wnt/β-catenin signal pathway in ICC cells. **A** Western blot analysis of expression of proteins related to Wnt/β-catenin signal pathway in HCCC-9810, RBE and QBC-939 cells treated with bufalin or control. **B** The quantification of western blot. Protein levels were normalized to tubulin. **C-E** Cell localization and expression levels of β-catenin, CCND1 and p-β-catenin. All results were presented as the mean ± SD (n = 3). *p < 0.05, **p < 0.01

The Wnt/β-catenin signaling pathway plays a critical role in cancer, and aberrant Wnt/β-catenin signaling has been found to be closely associated with many aspects of cancer initiation, progression, and malignant transformation [38]. To further explore the potential mechanism underlying the inhibitory effect of bufalin on ICC cell viability and metastasis, we examined the expression levels of related proteins in the Wnt/β-catenin signaling pathway. Our results showed that bufalin treatment inhibited β-catenin, P-β-catenin, and CyclinD1 (CCND1) levels in HCCC-9810, RBE, and QBC-939 in a decreasing dose-dependent manner (Fig. 3A, B). In addition, we also found that β-catenin, P-β-catenin, and CCND1 were localized in the cytoplasm and nucleus and showed dose-dependent decreasing fluorescence intensity (Fig. 3C–E). These findings suggest that bufalin may inhibit the Wnt/β-catenin signaling pathway in ICC cells.

### SKL2001 reversed the inhibitory effect of bufalin on the proliferation and migration of ICC

**Fig. 4:**
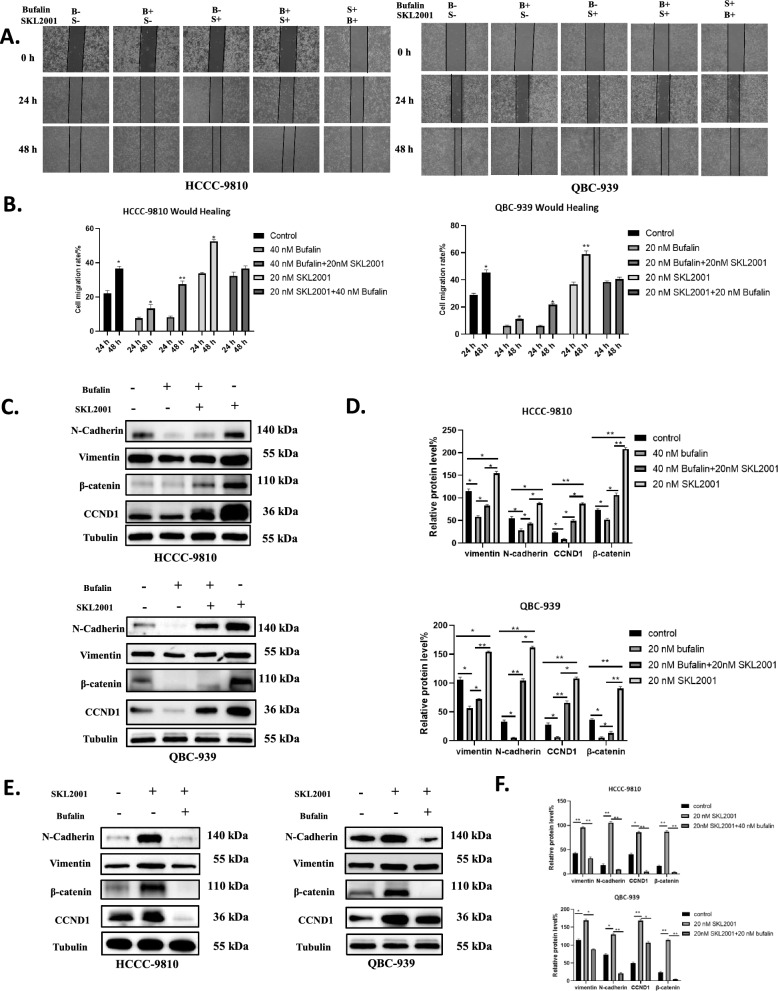
SKL2001 reversed the inhibitory effect of bufalin on the proliferation and migration of ICC. **A** In HCCC-9810 and QBC-939 cells, SKL2001 was activated after treatment with bufalin to detect the migration ability of the cells. After activation of SKL-2001, the cells were treated with bufalin to detect their ability to migrate. B above S indicates treatment with bufalin first, and S above B indicates treatment with advanced SKL-2001 activator. **B** Statistical analysis of (A) wound healing experiment. C In HCCC-9810 and QBC-939 cells, SKL2001 was activated after treatment with bufalin, and the expression levels of vimentin, N-cadherin, β-catenin and CCND1 were detected. **D** Statistical analysis of protein level of C.** E** The expression levels of vimentin, N-cadherin, β-catenin and p-β-catenin were detected in HCCC-9810 and QBC-939 cells after activation of SKL-2001 and treated with bufalin. **F** Statistical analysis of protein level of E. All results were presented as the mean ± SD (n = 3). *p < 0.05, **p < 0.01

SKL2001 is a small compound that has been found to be an activator of β-catenin [39]. To confirm that bufalin can inhibit ICC progression in a Wnt/β-catenin pathway-dependent manner, we performed an activator rescue assay of β-catenin on HCCC-9810 and QBC-939 cells inhibited by bufalin. As expected, after bufalin inhibited the proliferation and migration of ICC cells, the proliferation and migration of SKL2001 cells were significantly enhanced (Fig. 4A, B). In addition, we also verified that the proliferation and migration ability of ICC cells was enhanced after SKL2001 was added first, and this effect was inhibited after bufalin was added later (Fig. 4A, B). After bufalin inhibited ICC proliferation and migration, we found that the expressions of β-catenin and CCND1, N-cadherin and vimentin were increased after SKL2001 treatment. The addition of SKL-2001 alone significantly increased the expression of β-catenin, CCND1, N-cadherin, and vimentin compared to the control condition (Fig. 4C, D). In contrast, in ICC cells, activation of β-catenin with SKL-2001 resulted in significantly reduced expression of β-catenin, CCND1, N-cadherin, and vimentin after treatment with bufalin (Fig. 4E, F).

### Bufalin reversed the metastatic promotion induced by CAMKK2 in vitro

**Fig. 5:**
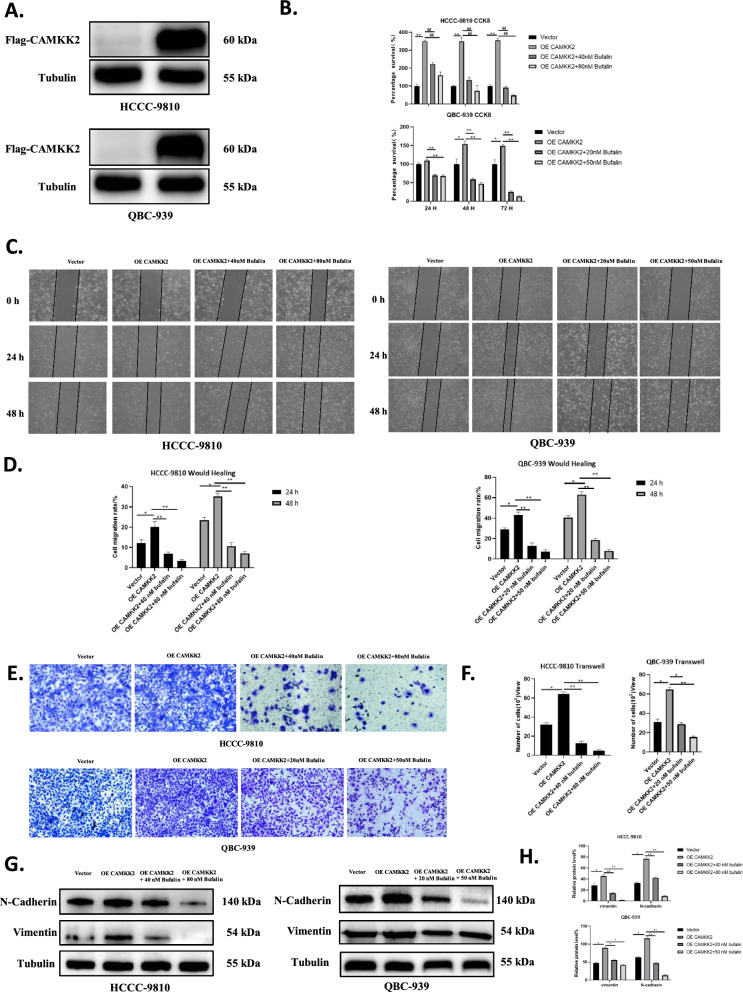
Bufalin reversed the metastatic promotion induced by CAMKK2 in *vitro*. **A** Western blot analysis of expression of Flag-CAMKK2 protein in HCCC-9810 and QBC-939 cells overexpressing CAMKK2 or vector. **B** CCK8 assay in HCCC-9810 and QBC-939 cells overexpressing CAMKK2 exposed to bufalin or vector. **C** Would healing in HCCC-9810 and QBC-939 cells overexpressing CAMKK2 exposed to bufalin or vector. **D** Statistical analysis of the cell migration in the wound healing assays. **E** Transwell in HCCC-9810 and QBC-939 cells overexpressing CAMKK2 exposed to bufalin or vector. **F** Statistical analysis of the cell migration in Transwell. **G** Western blot analysis of expression of metastasis related proteins N-cadherin and Vimentin in HCCC-9810 and QBC-939 cells. **H** The quantification of western blot (F). Protein levels were normalized to tubulin. All results were presented as the mean ± SD (n = 3). * p < 0.05, **p < 0.01

To investigate the effects of CAMKK2 on the proliferation and migration of ICC cells, we overexpressed CAMKK2 and transfected HCCC-9810 and QBC-939 cells with virus. Exogenously expressed CAMKK2 carried flag markers. We constructed cell lines stably transfected with overexpression of CAMKK2, and verified the overexpression of CAMKK2 in HCCC-9810 and QBC-939 through western blotting (Fig. 5A). The proliferation capacity of ICC was significantly enhanced after overexpression of CAMKK2, and this promotion effect was reversed by bufalin treatment (Fig. 5B). Similarly, the migration capacity of ICC was significantly enhanced after CAMKK2 overexpression, which was reversed by bufalin treatment (Fig. 5C–F). Western blotting was used to detect the expression levels of transfer-related proteins in HCCC-9810 and QBC-939 cell lines overexpressing CAMKK2. The results showed that overexpression of CAMKK2 led to increased expression of N-cadherin and vimentin in two ICC cell lines (Fig. 5G–H). In addition, bufalin therapy reversed the changes in protein expression levels caused by CAMKK2 overexpression. These results suggest that the overexpression of CAMKK2 in HCCC-9810 and QBC-939 cell lines can lead to the enhanced proliferation and migration of ICC. Overall, bufalin can reverse CAMKK2-induced metastasis promotion in vitro.

### Bufalin inhibits CAMKK2-induced Wnt/β-catenin signaling pathway in vitro

**Fig. 6:**
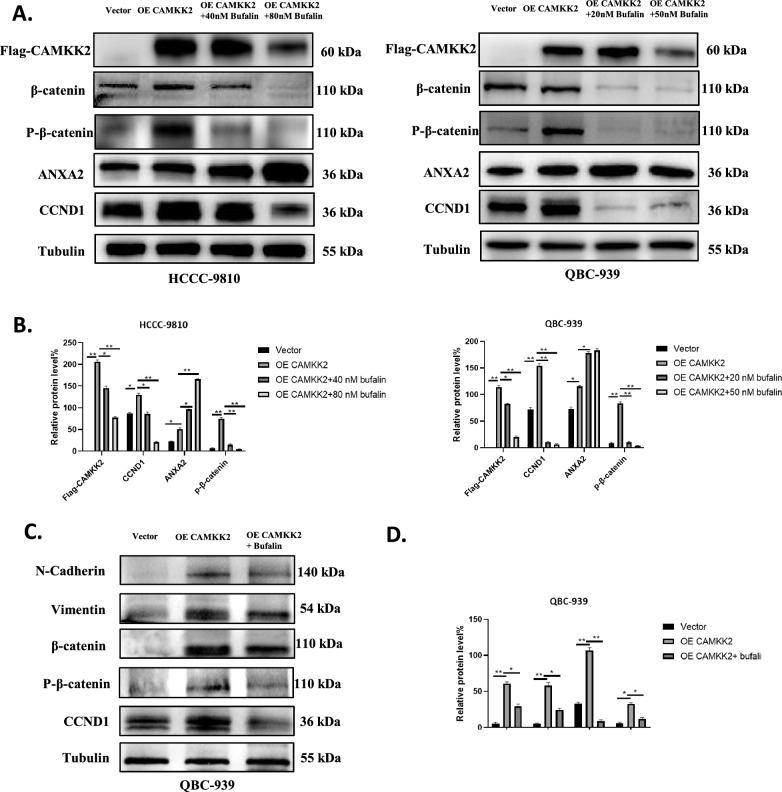
Bufalin inhibits CAMKK2-induced Wnt/β-catenin signaling pathway in *vitro* and *vivo*. **A** Western blot analysis of expression of proteins related to Wnt/β-catenin signal pathway in HCCC-9810 and QBC-939 cells overexpressing CAMKK2 or not, exposed to bufalin or not.** B** The quantification of western blot (A). Protein levels were normalized to tubulin. **C** Western blot analysis of expression of proteins related to vimentin, N-cadherin and Wnt/β-catenin signal pathway in solid tumors. **D** The quantification of western blot (C). Protein levels were normalized to tubulin. All results were presented as the mean ± SD (n = 3). * p < 0.05, **p < 0.01

To examine whether bufalin affects the Wnt/β-catenin signaling pathway through CAMKK2 in vitro, we used Western blotting to detect Wnt/β-catenin signaling pathway-related proteins in the HCCC-9810 and QBC-939 cell lines that overexpressed CAMKK2. The results showed that overexpression of CAMKK2 increased the expression levels of β-catenin, p-β-catenin and CCND1 proteins in *vitro*. At the same time, we treated two ICC cell lines overexpressing CAMKK2 with bufalin. Bufalin inhibited CAMKK2-induced Wnt/β-catenin signaling pathway associated protein levels (Fig. 6A, B). These results suggest that bufalin reverses the CAMKK2-induced activation of the Wnt/β-catenin signaling pathway in vitro.

### Bufalin reversed tumor‑promoting effect induced by CAMKK2 in vivo

**Fig. 7:**
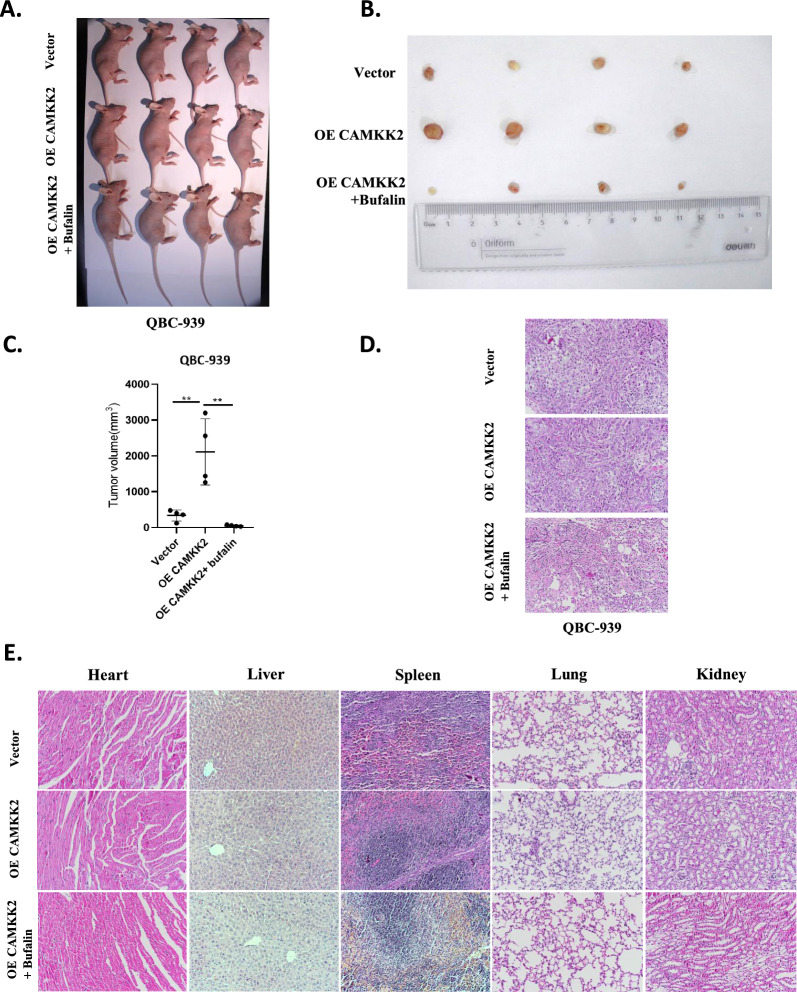
Bufalin reversed tumor‑promoting effect induced by CAMKK2 in *vivo*. **A** Tumor-bearing mice and the dissected tumors were photographed and shown. **B** A ruler was used to indicate the size of the tumors. **C** Statistics and comparison of tumor volume in overexpression group, bufalin treatment group and vector. **D** HE staining was used to detect the tumors. **E** HE staining of main organs in nude mice. Representative HE staining photos of heart, liver, spleen, lung and kidney of each group of nude mice. Bufalin had no obvious side effects on heart, liver, spleen, lung and kidney of nude mice. Protein levels were normalized to tubulin. All results were presented as the mean ± SD (n = 3). * p < 0.05, **p < 0.01

To investigate whether bufalin can affect the occurrence and development of ICC in vivo through CAMKK2, we subcutaneously injected QBC-939 cells stably overexpressing CAMKK2 into nude mice for validation. Due to the non tumorigenicity of the HCCC-9810 and RBE cell lines, these two types of cells were discarded in the nude mouse tumorigenic experiment. After tumor formation, the treatment groups were injected intraperitoneally with bufalin. There was no toxicity effect of bufalin on normal liver cells of L02. In addition, in our research group, no toxicity was found after treatment of bufalin with L929 fibroblasts [33]. No death caused by bufalin injection was found in nude mice until they were killed. Therefore, the toxicity of bufalin was not detected before injecting it into nude mice. Subcutaneous tumor analysis showed that the average tumors volume of the nude mice in the CAMKK2 overexpression group were significantly larger than that of the control group. After treatment with bufalin, the tumor volumes were significantly reduced in the treatment group (Fig. 7A–C). The tumors were all solid tumors, and the tumors in the bufalin treatment group showed extensive necrosis (Fig. 7D). Western blotting was used to detect the expression of metastasis-related proteins in solid tumors. The results showed that overexpression of CAMKK2 increased the expression of N-cadherin and vimentin and that bufalin reversed the CAMKK2-induced changes in the expression of these proteins after treatment (Fig. 6C, D). In addition, HE staining of the hearts, livers, spleens, lungs, and kidneys of nude mice showed no significant side effects of bufalin (Fig. 7E). These results suggest that bufalin can reverse the tumor-promoting effects induced by CAMKK2 in vivo.

### Bufalin reversed the activation of Wnt/β-catenin signal pathway induced by CAMKK2 in vivo

To explore whether bufalin also affects the Wnt/β-catenin signaling pathway through CAMKK2 in vivo, we used Western blotting to detect proteins related to this pathway in solid tumors. The results showed that the expression levels of p-β-catenin, β-catenin, and CCND1 proteins were significantly increased in tumor tissues overexpressing CAMKK2, while the expression levels of p-β-catenin, β-catenin, and CCND1 proteins were decreased in the bufalin-treated group (Fig. 6C, D). These results indicate that bufalin reverses the CAMKK2-induced activation of the Wnt/β-catenin signaling pathway in vivo.

### Bufalin inhibits CAMKK2 by inhibiting ICC proliferation and migration via ANXA2 and promoting mitochondrial dysfunction

**Fig. 8:**
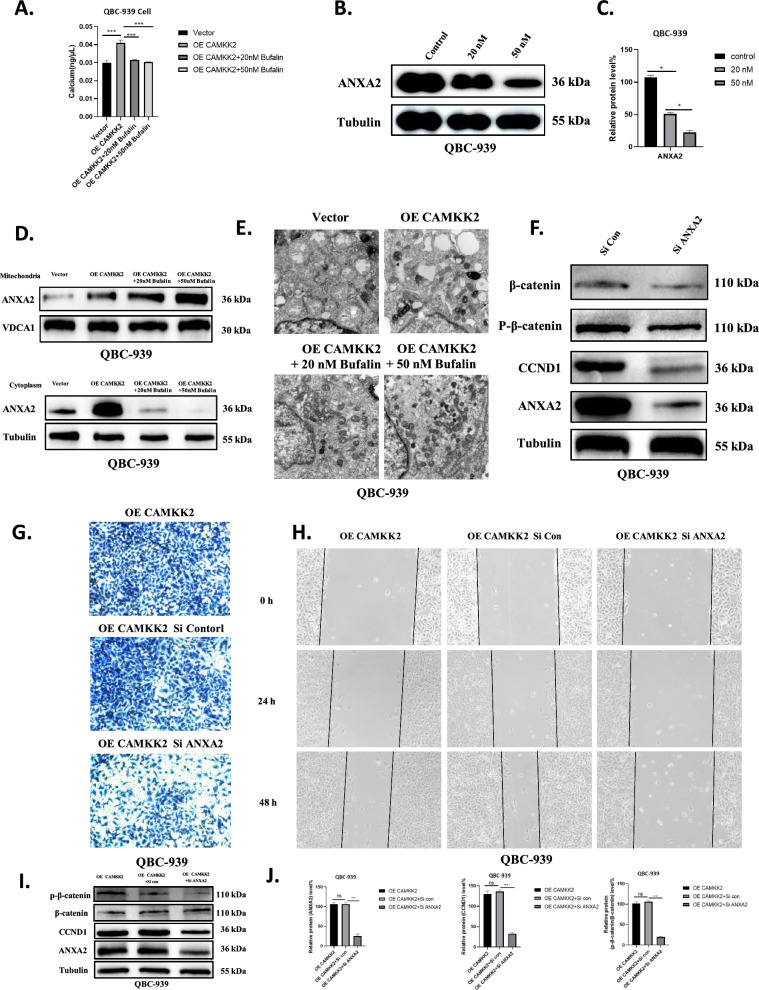
Bufalin inhibits CAMKK2 by inhibiting ICC proliferation and migration via ANXA2 and promoting mitochondrial dysfunction. **A** Ca^2+^ detection showed that after overexpression of CAMKK2, Ca^2+^ content increased, and after treatment with bufalin, Ca^2+^ content decreased. **B** Western blot analysis of expression of metastasis related proteins ANXA2 in QBC-939 cells exposed to bufalin or control. **C** The quantification of western blot. Protein levels were normalized to tubulin. **D** Mitochondrial protein and cytoplasmic protein were isolated, and the expression of ANXA2 in mitochondria and cytoplasm was detected. **E** Mitochondrial morphological changes after overexpression of CAMKK2 and treatment with bufalin were detected by transmission electron microscopy. **F** Expression of Wnt/ β-catenin signaling pathway related proteins after ANXA2 is knocked down in QBC-939 cells. **G-H** After overexpression of CAMKK2 in QBC-939 cells, ANXA2 is knocked down and the cell migration ability changes. **I** After overexpression of CAMKK2, ANXA2 is knocked down and Wnt/ β-catenin signaling pathway related proteins are expressed in QBC-939 cells. **J** Statistical analysis of protein level of I. All results were presented as the mean ± SD (n = 3). ns: p ≥ 0.05, *p < 0.05, **p < 0.01

We found that Ca^2+^ concentration increased after the overexpression of CAMKK2 and decreased dose-dependently after the addition of bufalin (Fig. 8A). Previous studies have shown that ANXA2 is a calcium-dependent phospholipid-binding protein. When intracellular calcium increases, ANXA2 is translocated to the cell membrane and then to lipid rafts, and turbulence can promote the intracellular calcium flow, resulting in the increase of intracellular calcium ions [40, 41]. The proliferation and migration of ICC cells were increased by the increase of intracellular calcium ions. In QBC-939 cells, ANXA2 expression decreased in a dose-dependent manner (Fig. 8B, C). Interestingly, we found that the expression of ANXA2 also increased after CAMKK2 overexpression, and we also found that bufalin-treated ICC cells overexpressing CAMKK2 with a dose-dependent increase (Fig. 6A). We isolated mitochondrial and cytoplasmic proteins and found that ANXA2 increased dose-dependently in mitochondria and decreased dose-dependently in cytoplasm (Fig. 8D). Transmission electron microscopy showed that the morphology of mitochondria in QBC-939 cells was slightly changed after overexpression of CAMKK2, and the morphology of mitochondria with added bufalin showed dose-dependent color enhancement and became irregular (Fig. 8E). After ANXA2 is knocked down, the expression levels of p-β-catenin, β-catenin, and CCND1 are significantly decreased (Fig. 8F). Therefore, we inferred that bufalin promoted mitochondrial dysfunction in ICC.

### Knocking down ANXA2 can reverse the proliferation and migration of ICC after overexpression of CAMKK2

**Fig. 9:**
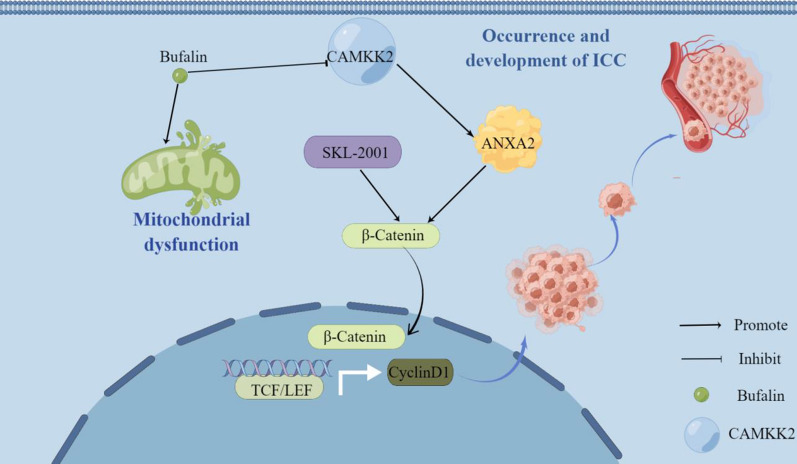
Bufalin targeting CAMKK2 affects ANXA2's inhibition of ICC occurrence and metastasis and promotion of mitochondrial dysfunction via the Wnt/ β-catenin signaling pathway. Bufalin down-regulates the expression of CAMKK2 in ICC, and then down-regulates ANXA2, reversing the activation of Wnt/β-catenin signaling pathway induced by CAMKK2/ANXA2, inhibiting the occurrence and metastasis of ICC, and promoting mitochondrial dysfunction. The figure is drawn in Figdraw software (www.figdraw.com)

After overexpression of CAMKK2, knock-down of ANXA2 significantly weakened the migration ability of QBC-939 (Fig. 8G). Wound healing experiments showed that after overexpression of CAMKK2, knockdown of ANXA2 was performed, and the migration ability of QBC-939 cells was reduced (Fig. 8H). Western blotting showed that the expression of N-cadherin and vimentin in QBC-939 decreased after overexpression of CAMKK2 and knockdown of ANXA2. The expression levels of β-catenin, p-β-catenin, and CCND1 also decreased (Fig. 8I, G). These evidences suggest that knockdown of ANXA2 can reverse the enhancement of ICC proliferation and migration induced by overexpression of CAMKK2. Finally, we show the whole results with a schematic figure (Fig. 9).

## Discussion

More and more evidence confirms that proliferation, migration and mitochondrial energy metabolism play key roles in tumor progression, and inhibiting proliferation, migration and promoting mitochondrial dysfunction in cancer is a promising strategy for tumor therapy [42]. Proliferation, migration and mitochondrial energy metabolism in tumor tissues is a multidimensional process co-regulated by tumor cells with various tumor-associated stromal cells as well as TME [43]. We found that bufalin could reverse proliferation, migration and mitochondrial energy metabolism induced by CAMKK2. Interestingly, related studies reported that overexpression of CAMKK2 caused an increase in GAPDH protein levels, so we discarded GAPDH and chose tubulin as our reference [44].

Bufalin is an anti-tumor active ingredient extracted from the venom of the traditional Chinese medicine toad poison. In terms of antitumor, bufalin inhibits tumor cell growth, promotes tumor cell differentiation and induces tumor cell apoptosis [45]. It was found that bufalin could inhibit the invasion and migration of bladder cancer cells by the mechanism of inhibiting the invasion of bladder cancer cells by decreasing blocking proteins, increasing transmembrane resistance and tightening tight junctions, and inhibiting their migration by activating the ERK pathway [46]. Bufalin inhibits the proliferation, migration, invasion and adhesion of hepatocellular carcinoma cells. The main mechanism is that bufalin significantly reduces the levels of p-AKT, p-GSK-3β, MMP-9 and MMP-2, increases the expression of GSK-3β and E-cadherin, and inhibits the nuclear translocation of β-catenin [47]. Moreover, bufalin can inhibit gastric cancer development and progression through PI3K/AKT/mTOR signalling pathway via BFAR [33]. The above studies have shown that bufalin can inhibit the proliferation, migration and apoptosis of tumor cells through multiple pathways. In the present study, we found that bufalin not only inhibited the proliferation, migration and invasion of ICC cells in vitro, but also inhibited the tumorigenesis and growth of QBC-939 cells in vivo. Bufalin reversed CAMKK2-induced Wnt/β-catenin signaling pathway activation both in vivo and in vitro. This finding suggests that bufalin could target CAMKK2 through this pathway, thereby exerting an inhibitory effect on ICC occurrence and metastasis. Past studies have found that CAMKK2 is regulated by Ca^2+^, and an increase in Ca^2+^ content activates the related expression of CAMKK2 [48]. Our study found that bufalin inhibited the decline in CAMKK2 protein expression, and we speculated that bufalin would inhibit the expression of Ca^2+^. Interestingly, this is the same as our test. Previous studies have shown that elevated Ca^2+^ content causes ANXA2 to bind to cell membranes and further promote Ca^2+^ inflow [41]. We found that bufalin inhibits Ca^2+^ levels. A decrease in Ca^2+^ levels causes ANXA2 to fall off the cell membrane, thereby blocking Ca^2+^ inflow and resulting in a further decline in Ca^2+^ content. Therefore, we suggest that bufalin inhibits CAMKK2 and affects ANXA2, subsequently impacting Wnt/β-catenin signaling pathway, promoting mitochondrial dysfunction, and inhibiting ICC proliferation and migration.

Our study has some limitations that need to be addressed in future studies. First, our study focused on the role of CAMKK2 in ICC cell proliferation and migration, but did not explore its effects on other aspects of ICC biology, such as cellular autophagy, angiogenesis, and immune responses. Second, our study screened bufalin as an inhibitor of CAMKK2 but did not evaluate its pharmacokinetic and pharmacodynamic properties, as well as its synergistic or antagonistic effects with other anti-ICC drugs. What’s more, bufalin’s drawbacks such as poor water solubility, low oral availability and short half-life limit its clinical application. Therefore, improving the pharmacokinetic profile of bufalin, prolonging its duration of action, increasing its efficacy and reducing its toxic side effects are essential for expanding its clinical applications. This is also a challenge that needs to be solved in the future.

In conclusion, this study confirmed the inhibitory effect of bufalin on the occurrence and metastasis of ICC. We screened CAMKK2 and determined it to be a protein that directly interacts with bufalin. We demonstrated the reversal of CAMKK2-induced ICC initiation and metastasis using bufalin in vivo and in vitro. We found that CAMKK2 can influence the morphology of mitochondria by influencing ANXA2 through changes in Ca^2+^. Finally, we demonstrated that bufalin reverses CAMKK2 to induce Wnt/β-catenin signaling pathways both in vivo and in vitro. These new results will provide new evidence for bufalin as a promising treatment for ICC.

### Supplementary Information

**Additional file 1.** An animal ethics certificate.

## Data Availability

The datasets used and/or analyzed during the current study are available from the corresponding author upon reasonable request. The data analyzed in this study were obtained from TCGA.

## Abbreviations

ICC: Intrahepatic cholangiocarcinoma
Ca^2+^: Calcium ion
CAMKK2: Calcium/calmodulin-dependent protein kinase kinase 2
ANXA2: Annexin A2
BLI: Bio-Layer Interferometry
siRNA: Silenced RNA
CCND1: CyclinD1
AR: Androgen receptor
IF: Immunofluorescence
IHC: Immunohistochemistry
GSEA: Gene Set Enrichment Analysis
BFAR: Bifunctional Apoptosis Regulator
GAPDH: Aldehyde-3-phosphate dehydrogenase
